# Supplementary material for: Optical clocks at sea
Source: Nature. 2024 Apr 24;628(8009):736–40. doi: 10.1038/s41586-024-07225-2 (PMC11043038; doi:10.1038/s41586-024-07225-2)
Supplement: Supplementary file 1 — Supplementary Information [file 41586_2024_7225_MOESM1_ESM.docx]

# Supplemental Material

## Potential Correlations in At-Sea Data

The acceleration sensitivity for VIPER was measured on land at < 1×10^-14^/g. For timescales relevant to ship dynamics (timescales < 10^3^ s), scaling the measured acceleration in Figure 3E by this coefficient implies that acceleration-induced shifts are ~100× below the clock noise. Timescales from 10^3^ – 10^5^ are more challenging to analyze and contain regions where temperature variation is strongest. Using independent environmental chamber testing on land, temperature coefficients were measured for PICKLES (< 5×10^-15^/°C), EPIC (2×10^-14^/°C), and VIPER (8×10^-14^/°C). Given this, common mode temperature fluctuations between clocks are relatively small, and the correlation between the Conex temperature fluctuations and clock instabilities is consistent with the measured temperature coefficients. Finally, measured drift rates consistent with the NIST measurements suggest that the instabilities for > 10^5^ s timescales accurately reflect the intrinsic behavior of the clocks. Notably, at the level that correlations could exist, cesium beam clocks are not precise enough to identify them on these timescales (e.g., Figure 1, Green trace).

With respect to magnetic fields, VIPER’s sensitivity has been measured at < 10^-14^/Gauss despite having no shield. EPIC and PICKLES include shielding which reduces the impact of Earth’s field by > 10×. Given the scale of the shifts and differences in the clock hardware, correlated magnetic sensitivity during the underway is very unlikely at these performance levels. Finally, no humidity-correlated frequency instabilities were observed among the three clocks despite ~15% changes over the ~10-minute AC period and 4-5% over day-night cycles throughout the underway.

## Operation of Clocks on Mobile Platforms

While optical clocks have been demonstrated outside of the laboratory [1], their use on moving platforms has been limited. While not as accurate as leading laboratory clocks, vapor cell clocks such as iodine offer practical advantages and enable state-of-the-art performance in mobile applications. Vapor spectroscopy operates continuously in an iodine clock, with high bandwidth (> 10 kHz), low orientation shift (~10^-14^/g), and without a local oscillator (LO) flywheel in the system.  Continuous operation eliminates the influence of noise aliasing through the Dick effect [2], which can put stringent requirements on the LO used in pulsed atomic clocks, especially under orientation changes and acceleration. Pulsed systems using conventional quartz LOs (~10^-10^/g) or rigidly mounted optical cavities (~10^-11^/g [3] and [4]) are difficult to field on mobile platforms without sacrificing performance by increasing the atom servo bandwidth. Moreover, orientation changes pose significant challenges for many spectroscopic approaches (e.g., free falling cold atoms, or ultra narrow spectroscopy of trapped atoms/ions that rely heavily on LO performance), resulting in practical barriers for use outside the laboratory. Compared to existing commercial clocks that do operate on mobile platforms (e.g., Cs beam, RAFS), iodine offers a significant performance advantage and reduction in environmental sensitivity (e.g., magnetic fields, temperature). With adequate servo bandwidth above the platform dynamics, it is expected that iodine clocks can be deployed on land, sea, air, and space platforms with similar performance to the results presented here.

Given current performance and SWaP, these clocks can already find near-term use in GNSS ground stations. Future space operation will require an order-of-magnitude reduction in SWaP, and radiation tolerance. We are actively developing a 5 L clock based on the same core physics package. These clocks share much of the iodine cell and laser componentry used in past [5] [6] and future space missions [7], thus presenting no fundamental barriers to space operation.

| [1] | M. Gellesch, J. Jones, R. Barron, A. Singh, Q. Sun, K. Bongs and Y. Singh, "Transportable optical atomic clocks for use in out-of-the-lab environments," *Advanced Optical Technologies,* vol. 9, no. 5, pp. 313-325, 2020. |
| --- | --- |
| [2] | G. J. Dick, "Local oscillator induced instabilities in trapped ion frequency standards," in *Proceedings of the 19th Annual Precise Time and Time Interval Systems and Applications Meeting*, 1989. |
| [3] | S. Webster and P. Gill, "Force-insensitive optical cavity," *Optics letters,* vol. 36, no. 18, pp. 3572-3574, 2011. |
| [4] | D. R. Leibrandt, M. J. Thorpe, J. C. Bergquist and T. Rosenband, "Field-test of a robust, portable, frequency-stable laser," *Optics Express,* vol. 19, no. 11, pp. 10278-10286, 2011. |
| [5] | W. Kokuyama, K. Numata and J. Camp, "Simple iodine reference at 1064 nm for absolute laser frequency determination in space applications," *Applied optics,* vol. 49, no. 32, pp. 6264-6267, 2010. |
| [6] | K. Döringshoff and et al., "Iodine frequency reference on a sounding rocket," *Physical Review Applied,* vol. 11, no. 5, p. 054068, 2019. |
| [7] | F. Kuschewski and et al., "COMPASSO mission and its iodine clock: outline of the clock design," *GPS Solutions,* vol. 28, no. 1, p. 10, 2024. |
